# The *h-*index in medical education: an analysis of medical education journal editorial boards

**DOI:** 10.1186/s12909-014-0251-8

**Published:** 2014-11-28

**Authors:** Asif Doja, Kaylee Eady, Tanya Horsley, M Dylan Bould, J Charles Victor, Margaret Sampson

**Affiliations:** Department of Paediatrics, University of Ottawa and Children’s Hospital of Eastern Ontario, 401 Smyth rd., Ottawa, ON K1H 8 L1 Canada; Children’s Hospital of Eastern Ontario Research Institute and University of Ottawa, 401 Smyth rd., Ottawa, ON K1H 8 L1 Canada; Research Unit, Royal College of Physicians and Surgeons of Canada, 774 Echo Drive, Ottawa, ON K1S 5 N8 Canada; Department of Anesthesiology, University of Ottawa and Children’s Hospital of Eastern Ontario, 401 Smyth rd., Ottawa, ON K1H 8 L1 Canada; Institute for Clinical Evaluative Sciences and Senior Lecturer, Institute of Health Policy, Management and Evaluation, University of Toronto, G1 06, 2075 Bayview Ave., Toronto, ON M4N 3 M5 Canada; Children’s Hospital of Eastern Ontario, 401 Smyth rd., Ottawa, ON K1H 8 L1 Canada

**Keywords:** *h*-index, Academic productivity, Medical education journals

## Abstract

**Background:**

Disciplines differ in their authorship and citation practices, thus discipline-specific *h-*index norms are desirable. Thus the goal of this study was to examine the relationship between the *h*-index and academic rank in the field of medical education, and the differences in the *h*-index between MD’s and PhD’s in this field.

**Methods:**

Due to the absence of a formalized registry of medical educators, we sampled available editorial board membership (considered a proxy for identifying ‘career’ medical educators) to establish h-index values. These were determined using Web of Science (WoS) and Google Scholar (GS), and internet searching was used to determine their academic rank. The correlation between authors’ *h*-indices derived from WoS and GS was also determined.

**Results:**

130 editors were identified (95 full professors, 21 associate professors, 14 assistant professors). A significant difference was noted between the *h*-indices of full professors and associate/assistant professors (*p* < .001). Median h-indices equaled 14 for full professors (Interquartile range [IQR] =11); 7 for associate professors (IQR =7) and 6.5 for assistant professors (IQR = 8). *h*-indices of MD’s and PhD’s did not differ significantly. Moderate correlation between GS and WOS *h*-indices was noted R = 0.46, *p* < .001.

**Conclusions:**

The results provide some guidance as to the expected *h*-indices of a select group of medical educators. No differences appear to exist between assistant professor and associate professor ranks or between MD’s and PhD’s.

## Background

Academic productivity is a difficult concept to quantify precisely, yet it is important at many academic institutions, particularly with regards to promotion and tenure. Research publications contribute significantly to one’s academic productivity, with a number of possible ways to measure research publication productivity. The most basic measure is the simple count of the number of publications; however, this count fails to recognize the relative importance of individual papers (beyond the recognition that it was approved for publication through peer review). Conversely, looking at the number of times a paper has been cited fails to take into account the number of papers an individual has produced. One could also look at the impact factor (IF) of the journal an author’s paper was published in, although the limitations of the impact factor have been well documented in the literature [1,2]. The IF can be influenced by a number of factors unrelated to article quality, including being influenced by subject area, type of documents or length of the citation measurement window. The IF is relatively insensitive to the documenting the impact of publications in “slow evolving” disciplines [2]. Additionally, it is important to note that the distribution of citations within a journal are typically very skewed, with articles in the “most cited half” of articles in a journal being cited up to 10 times as often as the “least cited half” [1].

To help address the limitations of these approaches, the *h*-index was developed by Hirsch [3] in 2005. The *h*-index is defined as follows: a scientist has index *h* if *h* of his or her *Np* papers have at least *h* citations each and the other (*Np-h*) papers have ≤ *h* citations each. For example, an *h*-index of 5 means that a scientist has published 5 papers that each have at least 5 citations.

A strength of the *h*-index is that it evaluates two surrogate measures of research, quantity (evaluated by the number of publications) and quality (evaluated by the number of citations of publications), and presents them as a single number. The *h*-index is, therefore, little affected by researchers who publish a high volume of low-impact papers or those who only have a few, high-impact publications.

There are some well described disadvantages to the h-index [4]. It attempts to describe a scientist’s whole career in a single number and so is, at best, reductionistic [5]. The accumulation of citations is dependent on the time since publication and so to some extent the *h*-index is dependent on the length of a scientist’s career and a scientist’s *h*-index can only go up (or stay the same) over time, even if they are no longer active.

However, despite some criticisms, the *h-*index can serve as a measure of an individual scientist’s productivity. The *h*-index has been shown to correlate well with peer judgments of research performance. Bornmann [6], for example, found that the average *h*-index of accepted versus rejected applicants for biomedical science research fellowships differed significantly [5]. Many scientific disciplines have shown a correlation between *h*-index and academic rank. Specifically with regards to medicine, studies in anesthesiology, urology, radiation oncology and neurosurgery have documented the average *h*-index associated with academic rank [7-12]. A study focusing on anesthesiologists has also demonstrated that the *h-*index correlates with the number of grants received [13]. 

The *h*-index has also been shown to outperform other bibliometric methods with regards to research performance in general surgery [14] and the metric has been demonstrated to be relatively unaffected by self-citation [9]. As an example, among general surgeons in Ontario, mean (SD) *h* index by academic rank was lecturer 1.0 (1.8); assistant professor 2.9 (4.1); associate professor 7.3 (6.1); and full professor 23.1 (13.6) (14). In the field of neurosurgery mean *h* indices were 4.9 (95% CI 2.5–87.3) for assistant professors, 8.3 (95% CI 5.9–10.7) for associate professors, 10.1 (95% CI 7.7–12.5) for professors, and 14.8 (95% CI 12.5–17.2) for chair- persons [8].

For the past several years, concerns have been raised as to the best way to measure academic productivity in medical education [15-18]. Certainly the *h-*index could provide one such benchmark for measuring productivity. However, it has been noted that different scientific disciplines have different citation practices and thus different *h*-indices. Differing citation practices amongst different disciplines occur based on a number of factors: (i) the number of publications in the periodical literature for a discipline; (ii) the average number of authors per paper; (iii) the average paper length; (iv) the average number of papers per author over a given period of time; (v) the theoretical or experimental mix that characterizes each discipline; (vi) the average number of references per paper; (vii) the proportion of references that are made to other articles in the periodical literature; (viii) the percentage of internationally co-authored papers, or (ix) the speed at which the citation process evolves [19]. As such, one cannot apply one “standard” *h*-index benchmark across disciplines, [20,21].

As a result it becomes necessary for each discipline and each subspecialty within medicine, to develop its own h-index norms [20]. Additionally, it is useful for disciplines to establish validity evidence for the *h*-index for academic productivity in their field; this has been done in many studies by examining the association between the *h*-index and various academic ranks [7,8,13,14].

We note that in recent years there has been a proliferation of alternative bibliometric indices, each intended to address specific limitations in existing metrics, including but not limited to: but including the *g*-index (to give more weight to highly cited papers) [22]; the contemporary *h*-index [23] and the age-weighted citation rate (to account for the age of papers) [24]; the individual *h*-index [25] and the *h*m-index (to adjust for individual and multi-authored publications) [26]. However, none of these indices are as well established in healthcare as the *h*-index nor are they calculated automatically, as *h*-index is, by databases such as Scopus or the Web of Science. A discussion of these alternative indices, however, is outside the scope of this current study.

Medical educators are a heterogenous group consisting of MD’s who may be medical education researchers in addition to clinicians, and PhD researchers whose academic activities are focused primarily on medical education research. These groups, although studying the same field, may have differing amounts of time devoted to research and, as a result, may have differing *h*-indices. Because of this heterogeneity, when examining the *h*-index of “career” medical education researchers, it would be important to compare the *h*-indices of MD’s and PhD medical education researchers.

Additionally, many studies have found discrepancies between *h*-indices derived from paid databases such as Web of Science (WoS) and freely accessible databases, chiefly Google Scholar [9,27,28]. As such, ideally, when examining the *h*-index in a field, it would be advantageous to compare more than one database.

Thus, our study sought to answer the following questions: 1) For a select group of medical educators, is the h-index able to distinguish between academic ranks (*Primary Objective*)? 2) What is the strength of the correlation between the *h*-index derived from WoS and GS for a given individual (*Secondary Objective*) 3) Do differences exist between MD’s and non-MD’s with respect to the *h*-index at various academic ranks within the context of health professions education (*Secondary Objective*)?

## Methods

### Sample selection

Medical education consists of a very heterogeneous group of scholars including physicians, PhD researchers and allied health care professionals. As such, unlike medical specialties, there are no comprehensive or mandated registries of medical educators. Thus in our study we chose to use the members of editorial boards of leading medical education journals. The use of journal editorial board members has been used in other papers that examined the *h*-index in various medical fields [12,29-31]. Using editorial board members, allowed us to be relatively assured that the individuals studied would be considered established medical education scholars, including MD’s doing medical education research, non-MD’s who are medical education scholars and other health professionals. While we could simply have looked at authors who published articles in medical education journals, this would have been an excessively heterogeneous group; in addition to including individuals who dedicated their careers to medical education research, such a group would also include students and residents who may not be considered “career” medical education scholars per se, education scholars who do not primarily do medical education research and clinicians who may spend a minority of their time on medical education research.

In a previous study, we identified the medical education journals that are the most productive (i.e. publish the most number of evaluative original research medical education articles), namely *Academic Medicine, Medical Education, Medical Teacher, Teaching & Learning in Medicine and Journal of Continuing Education in the Health Professions* [32]; consequently, the editorial board members of these journals were included in the present study.

A list of editorial board members was generated by accessing the various journals’ websites. A web search of the names of all editorial board members was conducted to obtain the credentials, academic affiliation, academic rank, faculty/department/division, as well as any additional role(s) these identified authors may have (i.e., chair, director, associate dean, etc.). Academic rank was characterized as Assistant Professor (including adjunct professors or lecturers), Associate Professor, or Full Professor (including Professor Emeritus). Only the most up to date information was recorded and in any case where the academic rank could not be accurately determined, the individual in question was contacted by e-mail. The e-mail contained full disclosure of the intent of our study; consent was implied if they chose to respond to the email with their academic rank.

We also characterized scholars as to whether they were MD’s or non-MD’s (holders of PhDs, DPhil or Masters degrees without an MD or equivalent degree). MD’s who also held an advanced degree (i.e. those with a Masters or PhD) were still classified in the MD category. For the MD group, we also attempted to determine what proportion of their publications were medical education articles and what were other types of articles (i.e. clinical or basic science).

To evaluate the likely power of our study, the results of Bould et al. [10] were used. They found a large difference between full professors (h-index 18.0, SD 8.3) and associate professors (h-index 9.5, SD 6.5) – a Cohen's d effect size of 1.14. Fixing the probability of type-I error at 0.05, a non-parametric analysis (using the minimum asymptotic relative efficiency assumption) with a sample size of 16 per group would provide 80% power to detect such an effect. Bould et al. [10] found a smaller, but still large effect size between associate professors and assistant professors (h-index 4.2, SD 4.2) – a Cohen's d of 0.97. In this case, a sample size of 21 per group would provide 80% power (G*Power3, version 3.1.2, Düsseldorf, Germany).

### Determination of *h*-index

The *h*-index for all identified authors was determined using WoS. Although there are other databases that could have been used, they have significant limitations. The Scopus database is limited in that *h-*index calculations are only based on publications from 1996 to the present. Since many individuals we would be examining would be full professors, we expected that they may have highly cited papers published prior to 1996. Google Scholar (GS), while being advantageous because it is a free database, has been identified as having significant discrepancies when determining the *h*-index and citations, as compared to WoS and Scopus [9,27,28]. However as a secondary analysis, we did examine the correlations between the *h*-indices derived from WoS and GS.

The full name of the author including the middle initial, if known, was entered into Google Scholar, automatically generating the author’s *h*-index. WoS required the author’s last name and initials. Because many authors use variations of their name, the search was conducted first using the last name and all known initials, and then using the last name and the first initial. In every case, the “exact matches only” option was selected. The results of both searches were examined to determine the form of the author’s name that generated the most accurate results. Finally, as there may be more than one author with the same name, results were refined by selecting only the relevant Web of Science categories (for example, life science and biomedicine) to eliminate authors from other fields. From these results, WoS created a citation report indicating the author’s *h*-index.

### Statistical analysis

Due to the skewed nature of the *h*-index distribution in our data, non-parametric statistics were used for analysis. The median was used as the measure of central tendency and interquartile range (IQR) was used as the measure of dispersion. Overall comparison of *h*-indices across all academic ranks was performed with the Kruskal-Wallis one-way analysis of variance. Post-hoc comparisons of *h*-indices between academic ranks were performed with the Mann–Whitney U test using a Bonferroni correction to the p-value to account for multiple comparisons. Comparison of *h*-indices between MD’s and non-MD’s from various academic ranks was also performed using the Mann–Whitney U test. *h*-indices derived from WoS and GS were correlated using Spearman’s rank correlation coefficient.

Ethical Approval was obtained from the Children’s Hospital of Eastern Ontario Research Ethics Board on April 15, 2012 (File No 10001455).

## Results

Searches were conducted between December 2012 and January 2013 which produced 189 names. The initial sample of names was reviewed to ensure accuracy of data. All duplicate names (n = 27) and those without an academic affiliation (n = 13) were removed from the dataset. Those members whose academic rank could not be determined with accuracy were excluded from the study (n = 19). 130 editors were included within our final sample (95 full professors, 21 associate professors, 14 assistant professors) (Table 1).

**Table 1:** **Academic rank of editors retrieved via search, categorized by whether the researcher is an MD or non-MD (ie PhD, Dphil, Masters)**

**Academic rank**	**Designation non-MD**	**Designation MD**	**Total**
Professor	43	52	**95**
Associate Professor	7	14	**21**
Assistant	7	7	**14**
**Total**	**56**	**73**	**130**

**Figure 1:**
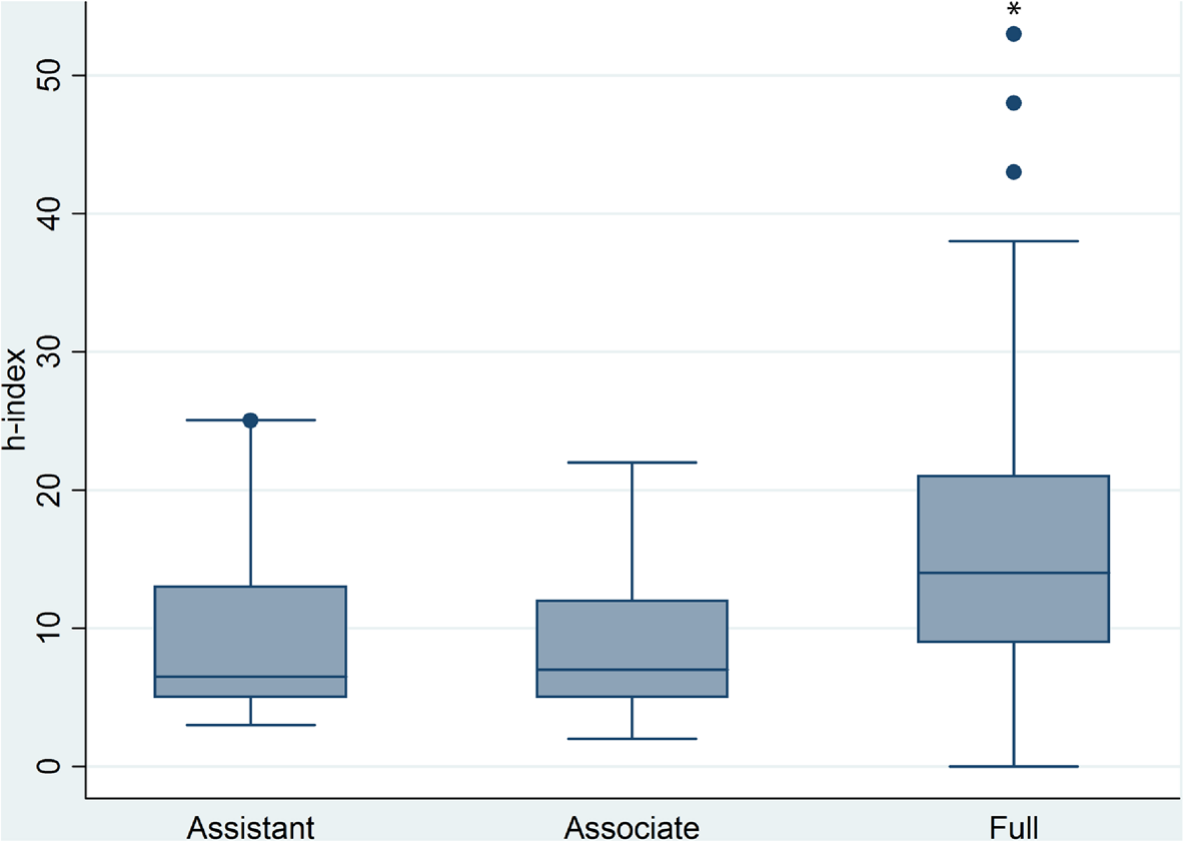
**Box plot of WoS** ***h*** **-index for each academic rank.** The height is the interquartile range (IQR). The heavy line is the median. The lower whisker extends to the lowest value within 1.5 IQR of the lower quartile, and the upper whisker extends to the highest value within 1.5 IQR of the upper quartile. Circles represent outliers. An asterisk represents a significant difference between groups.

Results showed a significant difference between the *h*-indices of full professors as compared to associate and assistant professor (H = 16.04; df = 2; *p* < .001) groups. Full professors had a median *h*-index of 14 (IQR = 11); associate professors had a median *h*-index of 7 (IQR = 7); and assistant professors had a median *h*-index of 6.5 (IQR = 8). Overall there was a significant difference between *h*-indices when comparing all academic ranks (*p* < .001). However, when performing individual comparisons amongst academic ranks, significant differences were only found when comparing the *h*-indices of full professors to associate (*p* = .001) and assistant professors (*p* = .005). No significant difference was found when comparing the *h*-indices of assistant professors to associate professors (*p* = .80) (Figure 1).

**Figure 2:**
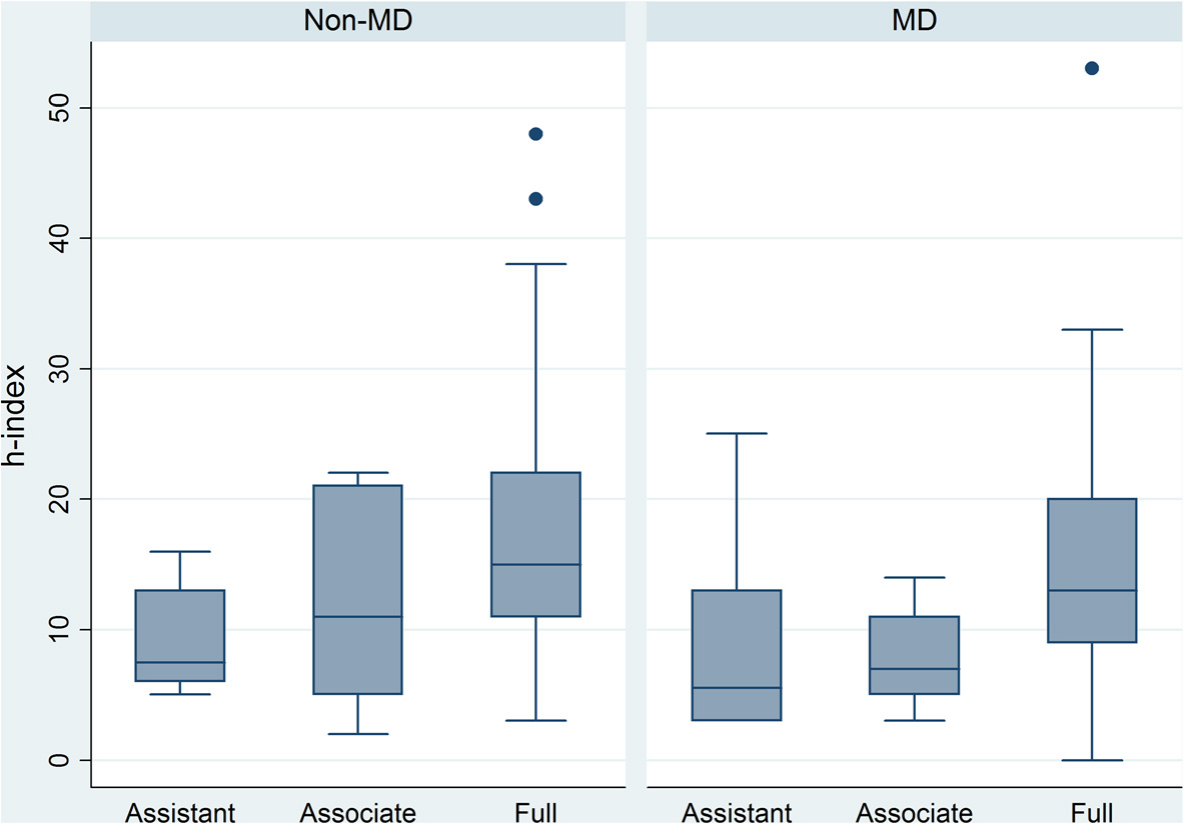
**Box plot of WoS** ***h*** **-index for each academic rank comparing MD’s and non-MD’s.** The height is the interquartile range. The heavy line is the median. The lower whisker extends to the lowest value within 1.5 IQR of the lower quartile, and the upper whisker extends to the highest value within 1.5 IQR of the upper quartile. Circles represent outliers. An asterisk represents a significant difference between groups.

No significant differences were found when comparing the *h*-indices of MD’s and non-MD’s at various academic ranks (Figure 2). When examining the MD group, the median proportion of articles which were medical education papers versus other papers was as follows: assistant professors 82% (IQR = 66%); associate professors 91% (IQR = 25%); and full professors 71% (IQR = 75%). Looking at all editorial board members, the correlation between WoS derived *h*-indices and GS derived *h*-indices was R = 0.46, *p* < .001.

## Discussion

Using editorial board members from medical education journals, we were able to determine the median *h*-index for various academic ranks. Several important points must be taken into consideration when examining our results. Editorial board members, by the very nature of the positions they hold, publish more frequently than the ‘average’ medical educator. As such, our results are intended to represent the *h*-indices for highly accomplished scholars at various ranks in medical education. The average *h* index of editors of selected otolaryngology journals was 15 [31], comparable to the full professors in our sample. As mentioned, among general surgeons in Ontario, mean *h* index by academic rank was lecturer 1.0 ; assistant professor 2.9; associate professor 7.3; and full professor 23.1 (14). In the neurosurgery mean *h* indices were 4.9 or assistant professors, 8.3 for associate professors, 10.1 for professors, and 14.8 for chair- persons. These differing results amongst different areas of medicine for various academic ranks underscores the difficulty of comparing *h*-indices between different fields.

One major finding was the lack of significant difference between the *h*-indices of assistant and associate professors in our sample. This may simply be due to the fact that we were underpowered to detect a difference between these two groups. However, this finding may also be due to the fact that these assistant professors, despite having not been promoted to associate professor at the time of our study, had a certain degree of academic productivity and/or were considered an “expert” in medical education in order to be offered editorial board membership. As such, it is reasonable to assume that editorial board members with a rank of assistant professor would have a relatively high *h*-index, more in line with associate professors.

We did note a large degree of variance in *h*-indices, particularly at the rank of full professor; this variation demonstrates the limitations of using the h-index alone to measure academic productivity. Academic rank is only a surrogate for research achievements and may also be influenced by alternatives to the research career track, including excellence in administration, education, and even clinical work. Further research investigating this variation may provide answers to whether we can further improve the assessment of research success beyond current subjective means and existing metrics.

We also noted no difference between MD’s and non-MD’s regarding *h*-indices for various academic ranks. This may seem counter-intuitive, as one would assume that the non-MD’s might have a higher *h*-index, as they conceivably could have more dedicated time for research. However, we noted that for all academic ranks, a substantial proportion of all publications in the MD group were non-medical education articles. Since the field of medicine as a whole is likely larger than the field of “medical education”, clinical medicine research papers may be more highly cited than medical education papers, and thus this may increase MD medical educators’ *h*-indices. This is an important caveat to consider when comparing MD’s and non-MD’s with respect to the *h*-index.

We noted only a moderate correlation between WoS and GS when it came to determining an individual’s *h*-index; however since both measures are purporting to measure the same thing, a very high correlation would be expected. Three sources of error that may have reduced this correlation will be considered [29]. First, papers by other authors may sometimes have been included in the *h-*indices of our editors. In this case, WoS allowed greater control through the ability to select fields of study. Second, there may be differences introduced by the breadth of coverage of the databases. We chose not to use Scopus as a data source since it used only articles published since 1996. Articles published in journals not indexed by WoS would not be included in WoS derived scores. GS may have the advantage over WoS in this regard due to its vast indexing base. Third, there may be database errors, i.e. where citations to another work are included in error. Franceschini & Maisano provide an example where numerous purported citations to a book predated the publication of the book (and were not advance reviews) [33]. WoS is thought to have an advantage over GS in terms of record linkage accuracy [34-36].

A significant problem in all bibliometric research, and with all the databases we examined, is the issue of name disambiguation – overcoming variability how authors may identify themselves and distinguishing between the works of authors with the same name. This may be problematic if scholars have a common name, have changed their name or use different initials in different publications. Institutional affiliation may provide some help, but authors may change institutions, and affiliations are most reliable for the first author. Various solutions have been put forth to this problem, including automated systems and author-maintained registries of their work. An example of the latter solution is the Système d’Interrogation, de Gestion et d’Analyse des Publications Scientifiques (SIGAPS), which relies on PubMed and is mandatory for all physicians in France [37]. Additionally, WoS has an automated system Distinct Author Identification System (DAIS) (http://images.webofknowledge.com/WOK46/help/WOS/h_da_sets.html) and an author-maintained system and GS also allows authors to create and maintain a profile. Research ID is another website which was developed to attempt to address name disambiguation (http://www.researcherid.com/).

Tang and Walsh [38] describe an automated system based on overlap of characteristics of referenced articles, weighted by overall citedness of those references, and the number of references in the articles. That is, not every cited reference has the same disambiguating potential. The underlying premise of such a system is that each author draws repeatedly from their knowledge base, and their reference creates a bibliometric fingerprint [36]. However, this approach is complex and may not perform well in narrowly defined subject areas, interdisciplinary work, or to span changing research interests across a career.

Some further study limitations should be noted. Much of academic productivity in medical education, including mentorship, teaching, curriculum development, program evaluation are often considered in decisions affecting promotion, whereas this information is not captured by the *h*-index. The small sample numbers in the assistant and associate professor groups are also a significant limitation and may have been responsible for lack of significant difference in the *h*-index between these groups.

Our data are reliant on websites and web searching to determine the academic ranks of editorial board members. As such, we may have been inaccurate in assigning academic ranks to some of the individuals in our study. However, as mentioned, when academic rank was in doubt we attempted to contact the professor to confirm academic rank, and excluded those whose academic rank could not be determined with certainty.

The most significant limitation to our study is the use of editorial board members as the population studied. As mentioned, this sample was chosen due to the fact that there are no easily accessible databases of “medical education scholars” compared to the readily accessible lists of editorial board members on journal websites. However we must bear in mind that we are looking at a very narrow, highly accomplished group of medical education researchers. As such it would be unfair to compare the *h*-indices of the individuals documented in this paper to the overall population of medical educators.

### Advice for promotion committees

Promotion committees hoping to use this data to help in decisions regarding academic promotion must be mindful of this particular limitation. We do not have the data to support an argument that failing to achieve an *h*-index of 14 for an aspiring full professor or 7 for an associate professor is grounds to not promote someone. Conversely however, meeting or exceeding these *h*-index values, should be seen as a strong supporting argument for promotion.

The variability of *h*-index, and problematic reliability depending on which database is used for calculation of the *h*-index, should also be considered when using the *h*-index for academic promotion. At a minimum, automatically computed scores should be considered only estimates of a scholars’ true *h-*index. Recommendations should be made only within institutions, with reference to whether the academic has a research, educational, administrative, or clinical focus, and with a consistent and transparent choice about which database is chosen to calculate the *h*-index. Perhaps even more preferable to databases would be to determine the *h*-index by obtaining a list of publications from the curriculum vitae of the individual in question and then performing an in-depth citation analysis.

## Conclusions

The results provide some guidance as to the expected *h*-indices of a select group of medical educators, with assistant and associate professors having an h-index in the 6–7 range and full professors having an *h*-index in the 14 range. No differences appear to exist between MD’s and PhD’s. Because of the limitations of automatically calculated *h*-index scores, it is recommended to use robust methods of authentication of a researcher’s publications – such as thorough citation analysis based on the individuals curriculum vitae – when using the *h*-index for decisions regarding tenure and promotion.

## Abbreviations

GS: Google Scholar
WoS: Web of Science
